# Mayten Tree Seed Oil: Nutritional Value Evaluation According to Antioxidant Capacity and Bioactive Properties

**DOI:** 10.3390/foods10040729

**Published:** 2021-03-30

**Authors:** Rosanna Ginocchio, Eduardo Muñoz-Carvajal, Patricia Velásquez, Ady Giordano, Gloria Montenegro, Germán Colque-Perez, César Sáez-Navarrete

**Affiliations:** 1 Departamento de Ecosistemas y Medio Ambiente, Facultad de Agronomía e Ingeniería Forestal, Pontificia Universidad Católica de Chile, Av. Vicuña Mackenna, Macul 4860, Chile; rginocch@uc.cl; 2 Center of Applied Ecology and Sustainability (CAPES), Pontificia Universidad Católica de Chile, Av. Vicuña Mackenna, Macul 4860, Chile; 3 Departamento de Química Inorgánica, Facultad de Química y de Farmacia, Pontificia Universidad Católica de Chile, Av. Vicuña Mackenna, Macul 4860, Chile; eamunoz1@uc.cl (E.M.-C.); pdvelasquez@uc.cl (P.V.); 4 Departamento de Ciencias Vegetales, Facultad de Agronomía e Ingeniería Forestal, Pontificia Universidad Católica de Chile, Av. Vicuña Mackenna, Macul 4860, Chile; gmonten@uc.cl; 5 Departamento de Ingeniería Química y Bioprocesos, Facultad de Ingeniería, Pontificia Universidad Católica de Chile, Av. Vicuña Mackenna, Macul 4860, Chile; gjcolque@uc.cl (G.C.-P.); csaez@ing.puc.cl (C.S.-N.); 6 Centro de Energía UC, Pontificia Universidad Católica de Chile, Av. Vicuña Mackenna, Macul 4860, Chile

**Keywords:** antioxidant capacity, DPPH, solvent extraction, nutrition, *Maytenus boaria*, ABTS

## Abstract

The Mayten tree (*Maytenus boaria* Mol.), a native plant of Chile that grows under environmentally limiting conditions, was historically harvested to extract an edible oil, and may represent an opportunity to expand current vegetable oil production. Seeds were collected from Mayten trees in north-central Chile, and seed oil was extracted by solvent extraction. The seed oil showed a reddish coloration, with quality parameters similar to those of other vegetable oils. The fatty acid composition revealed high levels of monounsaturated and polyunsaturated fatty acids. Oleic and linoleic acids, which are relevant to the human diet, were well represented in the extracted Mayten tree seed oil. The oil displayed an antioxidant capacity due to the high contents of antioxidant compounds (polyphenols and carotenoids) and may have potential health benefits for diseases associated with oxidative stress.

## 1. Introduction

In recent decades, the oil crop sector has been one of the most dynamic agricultural segments worldwide, with a 4.3% per annum (p.a.) growth rate compared with an average of 2.1% p.a. for all agriculture [1]. Worldwide production, consumption, and trade in this sector have increasingly become dominated by a small number of crops [2,3], as palm, soybean, and rapeseed oils have represented 82% of the total global oil crop production since 1990 (according to oil equivalent measurements). Secondary oil crops represent major elements of the food supply and food security in several countries, including sesame oil (e.g., in Sudan, Uganda, Ethiopia, and Myanmar), groundnut oil (e.g., in Sudan, Ghana, Myanmar, Vietnam, Senegal, the United Republic of Tanzania, and Benin), coconut oil (e.g., in the Philippines, Sri Lanka, Vietnam, and Mexico), olive oil (Mediterranean countries), and cottonseed oil (e.g., in Central Asia, the Sahel, Pakistan, Turkey, and the Syrian Arabic Republic) [1].

Vegetable oils are derived from the seeds and fruits of plants. Among the vegetable oils that are derived from seeds (seed oils), most are currently obtained from only a few commercially significant species (i.e., soybeans, sunflowers, rapeseed, flax, oil palm nuts, castor beans, groundnuts, cottonseed, and shea nuts) [4]. The most common energy-rich compounds contained in the endosperm or cotyledons of seeds are carbohydrates (starches) and fatty acids (oils) [4,5]. However, several plant species that grow under environmentally limiting conditions (i.e., arid and semiarid climates or nutrient-poor soils) worldwide that are not currently used as oil crops are known to feature oil-bearing seeds [6]. These crops may constitute an opportunity for expanding vegetable oil production to regions where crop production is not feasible, either currently or in the future, due to global climate change scenarios. Therefore, many indigenous tree species, which may be more resistant than current agricultural crops to limiting environmental factors (i.e., heat, water stress, salinity, frosts, and pests) but are not yet grown commercially, are becoming increasingly recognized as potentially valuable sources of vegetable oils [7], such as the Mayten tree (*Maytenus boaria* Mol.).

The Mayten tree is a medium-sized evergreen tree (up to 15 m in height) that is native to Chile, Argentina, Peru, and Brazil [8]. In Chile, the species has a wide geographic distribution (28° to 45° Southern latitude and 15 to 1800 msl) [9,10], presenting great adaptability to different site conditions, such as precipitation levels (mean annual values from 355 to 2351 mm), soil pH (neutral to acidic), and soil water availability (dry to saturated) [8]. These trees have been described as having high seed oil contents. Scattered historical information has suggested that this seed oil was extracted from seeds collected from wild trees and used for human consumption during Colonial times (from 1600 to 1810) in central Chile [11]. Large-scale seed oil extraction and exportation to France has been described during the 19th century [12], likely for lamp oil use [13]. However, scarce information is available regarding the amounts produced, the extraction methods used, and the specific physicochemical characteristics of this seed oil. According to the work of Vicente Bustillos, from 1846 [13], Mayten tree seeds are easy to grind and press (cold or heat press), and large amounts of oil can be obtained (approx. 25% of total weight); the oil is transparent, reddish-yellowish in color, featuring a bitter taste and a density similar to that of olive oil (specific weight of 0.92), and it begins to freeze at 4–5 °C. Recent literature has indicated high oil contents in the seeds of the Mayten tree (40%) [14], which may be used for cooking [15] and industrial purposes (i.e., as a substitute for linseed oil) [16]. However, none of these applications are currently in use.

The Mayten tree belongs to the *Celastraceae* family, a plant group known to be rich in secondary metabolites (i.e., sesquiterpenes and agafurans), some of which have been reported to demonstrate interesting pharmacological behaviors [17], antiseptic properties [18] or pesticidal activities [19]. Other chemical compounds (i.e., agafurans) that have been isolated from the Mayten leaves feature biopesticidal or natural insecticidal properties [20]. Most of the studies that have been performed to explore secondary metabolites and bioactive properties have examined vegetative aerial tissues (leaves and stems); however, no studies have examined seeds or seed oil [8,14,21,22]. Therefore, the primary objective of the present study was to perform a physicochemical characterization of Mayten tree seed oil, including the evaluation of antioxidant capacity and bioactive properties to determine the nutritional value.

## 2. Materials and Methods

### 2.1. Seed Materials and Seed Oil Extraction

Seeds from Mayten trees (Figure S1) were collected in north-central Chile (Elqui Valley, Santiago Metropolitan Park, and Quirihue) from February 2016 to April 2016. The seeds were hand-cleaned, allowed to air dry, and stored at 5 °C. Seeds were ground using an electric coffee grinder, and the oil was extracted with solvents (2:4:2 *w*:*v*:*v* ratio of seeds:methanol:chloroform), according to the method described by Bligh and Dyer [23]. The mixture of ground seeds and solvents was agitated at 200 rpm for 3 h. The method described by Bligh and Dyer was modified by adding water only in the second stage of the extraction. Distilled water and chloroform were then added (1.8:2.0:2.0, *v*:*v*:*v*, mixture:water:chloroform), by forming a ternary system, and the mixture was vacuum filtered through a 7 µm pore filter. Two phases were collected, the phase of methanol–water composition (top), and the chloroform–seed oil (bottom). The chloroform was evaporated using a rotary evaporator (40 °C for 30 min), and the seed oil was stored at 4 °C for 30 min in the dark (Figure S1).

### 2.2. Physicochemical Analysis

Determination of the seed oil color was performed according to the method described by Popa and Doran [24] using a colorimeter (WR10, DANSTLEE). Each extract was placed in a quartz cuvette for measurement. Coordinate values were obtained, where L represents lightness and varies between 0 (black) and 100 (white), a expresses red (+) or green (−), and b indicates yellow (+) or blue (−). Chroma (C°) and hue (H°) values were obtained from the following Equations:

(1) $$\;C{}^{\circ}=\sqrt{a^{2}+b^{2}}$$

(2) $$\;H{}^{\circ}=\arctan\frac{a}{b}$$

The seed oil density was determined using pre-calibrated volumetric flasks [25]. The peroxide index, which measures the initial oxidation state, is expressed in milliequivalent of oxygen per kilogram of oil. The iodine value indicates the degree of unsaturation, expressed in gram of iodine absorbed by 100 g of oil. Free acidity, is the percentage of free acid present in the oil, expressed in oleic acid percentage. Rancimet 743 measures the stability oxidation of the oil, corresponds to the induction period, expressed in hours, and thiobarbituric acid reactive substances (TBARS) measure malondialdehyde (MDA) present in the sample. All analyses were determined according to the American Oil Chemists’ Society (AOCS) standard method [26], as described by Petropoulus et al. (2020) [27].

### 2.3. Fatty Acid Profile

Gas chromatography (GC) coupled with a flame ionization detector (FID) was used according to the AOCS standard method [26]. The following standard measurements were used: butanoic acid (C4:0); caproic acid (C6:0), caprylic acid (C8:0); capric acid (C10:0); undecylic acid (C11:0); lauric acid (C12:0); tridecylic acid (C13:0); myristic acid (C14:0); pentadecylic acid (C15:0); palmitic acid (C16:0); margaric acid (C17:0); stearic acid (C18:0); arachidic acid (C20:0); japonic acid (C21:0); behenic acid (C22:0); tetrasanoic acid (C24:0); tetradecene acid (C14:1); pentadecylic acid (C15:1); palmitoleic acid (C16:1); heptadecene acid (C17:1); oleic acid (C18:1); timnodonic acid (C20:1n9); erucic acid (C22:1n9); tetrasaenoic acid (C24:1); linoleic acid (C18:2n6); γ-linoleic acid (C18:3n6); α-linoleic acid (C18:3n3); gondoic acid (C20:2n6); di-homo-γ–linoleic acid (C20:3n6); dihomolinolenic acid (C20:3n3); eicosatetranoic acid (C20:4n6); eicosapentaenoic acid (EPA) (C20:5n3); docosadienenoic acid (C22:2); and docosahexaenoic acid (DHA) (C22:6n3).

### 2.4. Lipidic Indices

The qualities of the lipids in the seed oil were determined by evaluating the ratio of polyunsaturated fatty acids (PUFAs) to saturated fatty acids (SFAs) [28]. Two indices of coronary heart disease risk—the atherogenic index (AI), used to obtain the standard cardiac risk estimation; and the thrombogenic index (TI), which indicates the tendency to form clots in the blood vessels—were calculated as functions of the composition of monounsaturated fatty acids (MUFAs), PUFAs, SFAs, and specific fatty acids [29], as follows:

(3) $$\;\mathrm{AI}=\frac{\left(C12:0\;+\;4\;\times \;C14:0\;+\;C16:0\right)\;}{\left(\mathrm{MUFA}\;+\;\mathrm{PUFA}\right)}$$

(4) $$\;\mathrm{TI}=\frac{\left(C14:0\;+\;C16:0\;+\;C18:0\right)}{\left(0.5\;\times \;\mathrm{MUFA}\;+\;0.5\;\times \;n-6\mathrm{PUFA}\;+\;3\;\times \;n-3\mathrm{PUFA}\;\times \left(\frac{n-3\mathrm{PUFA}}{n-6\mathrm{PUFA}}\right)\right)}$$

### 2.5. Extraction of Antioxidant Compounds

Seed oil was mixed with methanol and hexane (1:5:1, *v*:*v*:*v*, oil:methanol:hexane) and maintained in an ultrasonic bath at 20 Hz for 20 min at a room temperature bath [30]. The solution was then centrifuged at 4000 rpm for 20 min at room temperature. The mixture was stored at 4 °C for 1 h, and the supernatant was then filtered through a 0.22 µm membrane with a syringe filter for the antioxidant extraction.

### 2.6. Quantification of Phenols and Flavonoids, and Determination of Antioxidant Capacity

Total polyphenolic contents (TPC) were estimated using the Folin–Ciocalteu (FC) method based on the antioxidant extract from oil, as described by Velásquez et al. (2019) [31]. Gallic acid (GA) was used as the standard; therefore, the resulting values are expressed in mg GAE (100 g)^−1^ of seed oil.

Total flavonoid contents (TFC) were estimated using the aluminum chloride method based on the antioxidant extract from oil, as described by Velásquez et al. (2019) [31]. Quercetin (Q) was used as the standard, and results are expressed in mg QE (100 g)^−1^ of seed oil. Some specific phenolic acids and flavonoids, such as 4-hydroxy benzoic acid, apigenin, caffeic acid, catechin, chlorogenic acid, cinnamic acid, chrysin, coumaric acid, epigenin, ferulic acid, gallic acid, kaempferol, luteolin, pinocembrin, quercetin, resveratrol, rutin, sinapic acid, syringic acid, vanillic acid, and phytohormone abscisic acid were quantified through using ultra-performance liquid chromatography (UPLC)-tandem mass spectrometry (MS/MS), according to the method described by Giordano et al. (2019) [32], by interpolation from standard calibration curves.

Antioxidant capacity was determined using the ferric reducing antioxidant power (FRAP) and 2,2′-azino-bis-(3-ethylbenzothiazoline-6-sulfonic acid (ABTS) radical-stabilization methods, according to the methods described by Diniyah et al. (2020) [33]. The antioxidant extract was measured using a FeSO_4_ standard and is expressed in mg FeSO_4_/100 g of seed oil, whereas Trolox (T) was used as the standard for radical stabilization, which is expressed in T equivalents (TE 100 g)^−1^ of seed oil. The 2,2-diphenyl-1-picrylhydrazyl (DPPH) radical was also assessed as described by Diniyah et al. (2020) [33], with some modifications, using several antioxidant extract dilutions. A curve was generated comparing the inhibition percentage against the tested dilutions (logarithmic relationship), interpolating the half-maximal inhibitory concentration (IC_50_), which is expressed in g of seed oil per mL^−1^ of methanolic extract. The Agilent 8453 UV-visible spectrophotometer (Palo Alto, CA, USA) was used for these analyses.

### 2.7. Extraction and Quantification of Carotenoids and β-Carotene

A carotene extract was obtained through seed oil saponification, as described by Varzakas and Kiokias (2016) [34]. The quantification of total carotenes was performed according to Bihler et al. (2010) [35], using β-carotene as the standard and expressed as mg β-carotene/100 g of seed oil. Absorbance was measured using the Agilent 8453 UV-visible spectrophotometer (Palo Alto, CA, USA).

The carotene profile and β-carotene levels were obtained using a high-performance liquid chromatography (HPLC)-diode-array detector (DAD). A reverse phase C18 column was used (150 mm × 4 mm × 5 µm), with a mobile phase flow of 1.5 mL min^−1^. Methanol (A), water (B), and n-butanol (C) were used as solvents in the following concentration gradient: 0 min 3% A, 92% B, and 5% C; 4 min 0% A, 92% B, and 8% C; 18.1 min 3% A, 92% B, and 5% C, until 23 min. The carotene profile was measured at 440 nm. The β-carotene level was interpolated from the calibration curve of a 10–100 mg/L β-carotene standard.

### 2.8. Statistical Analysis

All analyses were realized in triplicate (*n* = 3), and the data were expressed as the mean ± standard deviation using the statistical software Minitab 19.

## 3. Results and Discussion

### 3.1. Seed Oil Extraction

A yield of 61.77 ± 8.24% *w*/*w* seed oil was extracted from Mayten tree seeds, which is a yield larger than those for seed oils extracted from sunflower (51.00% *w*/*w*), sesame (48,00% *w*/*w*), and pumpkin seeds (46.00% *w*/*w*) [36].

### 3.2. Physicochemical Characteristics

The seed oil extracted from Mayten tree seeds had a reddish coloration, according to the tone angle (H: 59.40°), which is categorized among the purple–red colors [37]. The oil had a lower yellow and a higher red color composition (Table 1) compared with palm oil (b* = 56.87–74.50; a* = 2.21–13.0) [37]. According to the clarity analysis, Mayten tree seed oil is closer to white, based on a 1 to 100 scale, and is slightly darker than palm oil [37].

The Blight and Dyer solvent extraction method allows for the extraction of high molecular weight lipophilic compounds that exist in a solid state at 25 °C. This method resulted in oil dispersion [37,38], with a density greater than 1 (1.06 ± 0.07 g mL^−1^); as reported in Table 1, this is a value similar to the density of pine oil (1.042–1.224 g mL^−1^) extracted using organic solvents [25]. The seed oil density value was higher than that reported for palm oil (1.06 g mL^−1^ versus 0.892–0.899 g mL^−1^); such a value could indicate the presence of high molecular weight compounds, such as carotenoids and fatty acid [38].

The peroxide value identified for the Mayten tree seed oil was 5.10 ± 0.18 meq O_2_/kg oil (Table 1), a value similar to that reported for olive oil [23], and was within the range reported for palm oil (1.0–10.0 mg eq O_2_ g^−1^ oil) [38]. In addition, this peroxide value is below that allowed by the Chilean Sanitary Regulations of Food for the year 2015 (10.0 mg eq O_2_ g^−1^ oil) and also lower than that obtained from the seeds of the maqui berry [23].

The iodine value of 57.63 identified for Mayten tree seed oil was similar to the value for palm oil (46.0–56.0), but higher than that for coconut oil (6.0–11.0) [38,39], showing a similar degree of unsaturation with these oils. The free acidity of Mayten tree seed oil was within the range identified for palm oil (3.7–5%, Table 1) and within the range allowed by the Chilean Sanitary Regulations of Food for the year 2015 [23].

The TBARS index of the Mayten tree seed oil had a lower value than those for avocado and olive seed oils [40]. When examining the oxidative stability under acceleration conditions (Rancimet analysis), the Mayten tree seed oil demonstrated a longer induction period (52.15 ± 2.15 h) than soybean oil (11.2 h) or extra virgin olive oil (24–26 h) [41]. According to the obtained physicochemical properties, Mayten tree see oil has values similar to other commercial oils indicating that it could be used for human consumption.

### 3.3. Fatty Acid Profile and Nutritional Quality

**Figure 1:**
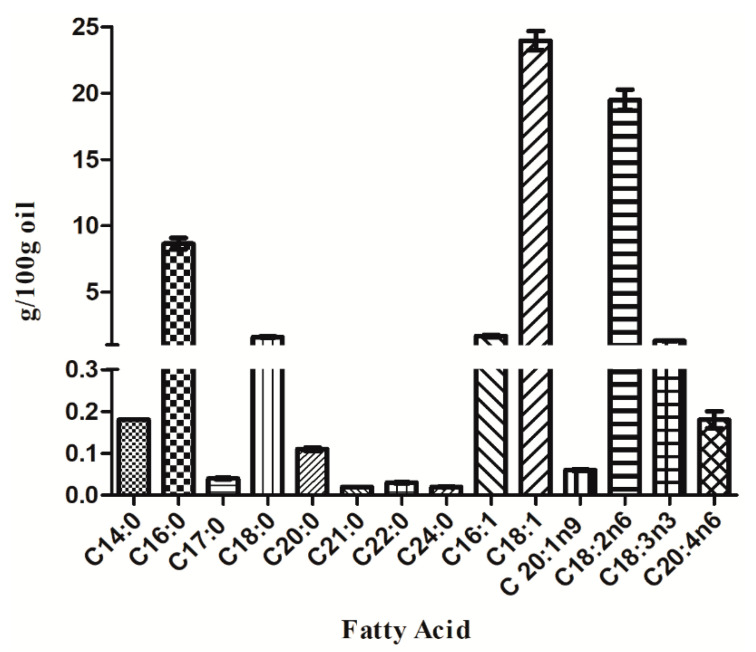
Profile of fatty acids of Mayten tree seed oil.

The fatty acid profile and nutritional qualities of Mayten tree seed oil were as shown in Figure 1 and Table 2, respectively. Mayten tree seed oil had a high unsaturated fatty acid content (Figure 1). The MUFA concentration of this seed oil (25.70 g/100 g oil) (Table 2) was lower than that for avocado seed oil (49–75.96%) [40,42,43], palm oil (37.1–39.2%) [38], olive oil (80.53%) [40], and extra virgin olive oil (71.32–79.62%) [41]. However, the PUFA composition of 20.98 g/100 g oil (20.98%) (Table 2) was greater than that for palm oil (8.1–10.5%) [38] and olive oil (5.43%) [40] and was within the range of that for avocado seed oil (11.75–37.13%) [40,42,43]. Based on these results, Mayten tree seed oils would likely provide health benefits, as unsaturated fatty acids favor specific enzymatic reactions such as cyclooxygenases, lipoxygenases and cytochrome P450 enzymes that resolve different processes of inflammation and protect against brain or renal dysfunctions [43,44], and PUFAs can provide the essential fatty acids that must be obtained through the diet.

The ingestion of foods with PUFA/SFA ratios in the range of 1.25 to 2.4 has been described to confer beneficial effects for the prevention of cardiovascular diseases (CVDs) [45]. As Mayten tree seed oil was found to have a PUFA/SFA value of 1.97 (Table 2), this oil would provide a comparative advantage over other commonly consumed seed oils, such as olive oil and menhaden oil [28].

The oleic acid (C18:1), linoleic acid (C18:2n6), and palmitic acid (C16:0) contents were high (in decreasing order) in Mayten tree seed oil (Figure 1). The oleic acid contents of Mayten tree seed oil were higher than in sunflower oil, similar to contents in soybean oil [46,47], and lower than in palm oil [38]. Oleic acid (C18:1) is an omega-9 MUFA with anti-thrombosis and other bioactive properties, which has been used in cosmetic and pharmacological applications [48].

The linoleic acid (C18:2n6) content in Mayten tree seed oil was higher than that in olive oil and palm oil [38]. Linoleic acid is often described as a precursor for other lipid mediators with anti-inflammatory properties [43]. Linoleic acid (C18:2n6) is an essential omega-6 fatty acid that has been shown to be involved in the maintenance of the skin’s permeable barrier against water, and the topical application of linoleic acid (C18:2n6) has been shown to improve dermatitis. Furthermore, linoleic acid is metabolized to arachidonic acid, which is a precursor of the eicosanoid compounds that regulate a range of physiological processes [49].

Palmitoleic acid (C16:1) and α-linoleic acid (C18:3n3) were found among the unsaturated fatty acid composition of Mayten tree seed oil (Figure 1), at higher levels than are found in sesame, sunflower, and rice brain [50]. Canola seed oil contains 3.4 g of palmitic acid per 100 g of oil [51], whereas the palmitic acid contents of Mayten tree seed oil were two-fold higher that in canola seed oil.

**Figure 2:**
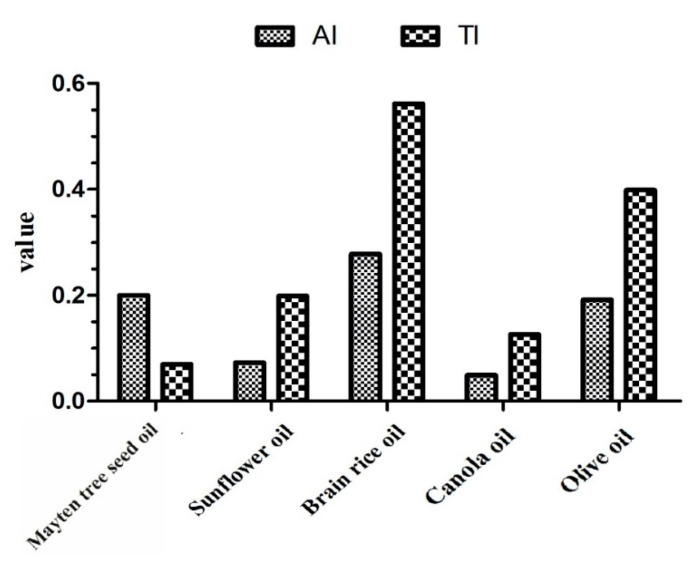
Atherogenic index (AI) and thrombogenic index (TI) values among Mayten tree seed oil and other commonly consumed seed oils (adapted from ref. [50]).

Two indices of coronary heart disease risk, AI and TI [29], were calculated for Mayten tree seed oil and other commonly consumed seed oils, as shown in Figure 2. The Mayten tree seed oil’s AI value was lower than for rice bran oil (Figure 2) and menhaden oil [28] but higher than for sunflower oil, canola oil, and olive oil (Figure 2). By contrast, the TI value for Mayten tree seed oil was lower than values found in other commonly used seed oils, such as olive, sunflower [28], rice bran, and canola oils [50] (Figure 2), likely due to the Mayten tree seed oil’s higher contents of oleic acid, a compound that reduces the risks of thrombosis [52]. These results indicate that Mayten tree seed oils could potentially reduce the risks of generating thrombi in blood vessels compared with other commonly consumed seed oils.

### 3.4. Polyphenol and Flavonoid Compounds

Polyphenol compounds (Table 3) were co-extracted with fatty acids during the seed oil extraction process from Mayten tree seeds, similar to other seed oils [23]. The TPC value (16.1 mg GAE/100 g oil) in Mayten tree seed oil represents 0.016% of the total oil contents, which is a larger proportion than is found in canola (11.26 mg GAE/100 g oil) [53] and olive oils (2–13 mg GAE/100 g oil) [54]. Flavonoids are a type of polyphenol component that dissolves in oil, and Mayten tree seed oil was found to contain a TFC of 15.5 mg EQ/100 g seed oil, which is similar to that reported for grape, rice bran, and chia seed oils but higher than those reported for sunflower, canola, soybean, and cottonseed oils [55].

Flavonoids, such as quercetin and myricetin; phenolic acids, such as ferulic, coumaric, and syringic acids; and the sesquiterpene abscisic acid (Table 3) were found in Mayten tree seed oil. Ferulic acid, the most abundant compound in mayten tree seed oil, has been demonstrated to display various pharmacological properties, including anti-inflammatory activities [56,57]. Ferulic acid has been shown to reduce cholesterol synthesis in the liver, followed by an increase in sterol acid secretion, and has been shown to act as a chemoprotective agent against coronary heart disease, preventing thrombi and sclerosis production [57,58]. Ferulic acid also inhibits the population growth of influenza, syncytial, and human immunodeficiency viruses [56] and has been shown to exert anticancer activity against colon and rectal cancer [57]. Similar to ferulic acid, the other polyphenolic compounds identified in this seed oil are known to provide health benefits, such as antioxidant properties against free radical formation and the prevention of diseases and infections due to antimicrobial activities [59].

### 3.5. Total Carotenoids and β-Carotene

The reddish coloration observed for Mayten tree seed oil was likely derived from its high carotene content (Table 3), which is present in the seed aril (Figure S1) [42,43]. This relationship between seed oil color and presence of carotenoids has also been described for palm and cucumber seed oils [60,61]. Mayten tree seed oil is darker in color because it has a total carotene content (TCC) that is three times higher than that of palm seed oil [38].

Approximately 70% of the total carotene content in Mayten tree seed oil is represented by β-carotene (Table 3). As a precursor of vitamin A, β-carotene plays an important role in the prevention of cataracts and macular hatching, in addition to improving night blindness and dry eyes [62]. The difference between the TCC and β-carotene contents in Mayten tree seed oil is expected to indicate the presence of other carotenoid compounds that were not specifically identified in the present study. The high carotene contents, together with the presence of polyphenols, may give this seed oil resistance to fatty acid peroxidation, which would prevent rancidity [63].

### 3.6. Antioxidant Capacity

The resistance against factors that cause rancidity in seeds oils is a function of the antioxidant capacity of seed oils, which can provide nutritional value without requiring the incorporation of synthetic antioxidants, such as those used by the food industry [64]. The antioxidant capacity of Mayten tree seed oil against the ABTS radical appeared to be lower than the capacities found in other seed oils (Table 4). However, this characteristic is typically determined based on the presence of polyphenolic compounds (as in the present study) and carotenoids because these molecules also react to the ABTS radical [65].

**Figure 3:**
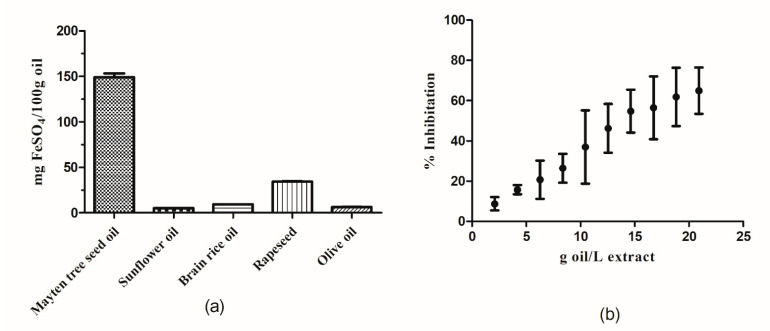
(**a**) Ferric reducing antioxidant power (FRAP) values for Mayten tree seed oil, sunflower oil, brain rice oil, canola oil, and olive oil. (Adapted with permission from Szydłowska-Czerniak, A. et al. [67], Copyright 2008 Elsevier); (**b**) Inhibition of the 2,2-diphenyl-1-picrylhydrazyl (DPPH) radical by Mayten tree seed oil. Values are represent as mean ± standard deviation.

The FRAP value is a measure of the antioxidant capability based on the evaluation of electron donation that occurs due to the activity of antioxidant compounds (Table 4) [66]. For Mayten tree seed oil, the FRAP value was three-fold higher that of butylhydroxytoluene (BHT), an artificial antioxidant commonly used by the food industry [64], indicating that Mayten tree seed oil displays a high antioxidant capacity. According to the IC_50_ value, a low amount of Mayten tree seed oil dissolved in methanol was necessary to inhibit 50% of the DPPH radical [64], indicating a better response against the proton donation mechanism of oxidated compounds. Based on the FRAP results, the antioxidant capacity of Mayten tree seed oil was found to be three-fold that of rapeseed oil and 15-fold those for sunflower, rice brain, and olive oils [50,67]. The percentage of DPPH radical inhibition of a decreasing methanol dilution range of Mayten tree seed oil is shown in Figure 3b. Initially, the percentage of inhibition increased as the contents of the seed oil increases, followed by a stabilization when 60–70% inhibition of the DPPH radical was achieved. Therefore, the examined Mayten tree seed oil appears to have a higher antioxidant capacity than the BHT supplement commonly used by the food industry (Figure 3).

## 4. Conclusions

Mayten tree seed oil was obtained through the application of the Blight and Dyer solvent (methanol–chloroform–water) extraction method, resulting in a 61.77% yield, which may be further improved using other extraction procedures. This seed oil was found to have high omega-6 and omega-9 fatty acid contents, with a higher PUFA content than that found in most major commercial vegetable oils.

The analyzed quality parameters indicated that Mayten tree seed oil has a high resistance against rancidity, with values similar to those of other commercial seed oils and within the requirements of Chilean Health Regulations. The high TPC and TFC values for this seed oil provide protection against lipid peroxidation and result in high ABTS, DPPH, and FRAP antioxidant capacities, which may be beneficial for human health and pharmacological uses.

The reddish coloration of Mayten tree seed oil is associated with a high content of carotenoids, which may constitute a comparative advantage relative to other vegetable oils currently used for human consumption. β-carotene is the dominant carotenoid in this seed oil, potentially providing elements that can contribute to the functional properties of this oil.

The results of the present study indicate that Mayten tree seed oil has nutritional value, based on the antioxidant capacity and bioactive properties identified for this oil. This seed oil would be an interesting alternative to other vegetable oils intended for human consumption, particularly since it could be produced in areas affected by global climate change with higher yields than other traditional oils. It could be considered as a functional food, a carotenoids supplement or an antioxidants additive ingredient for the food industry. However, studies exploring the domestication of this tree species are necessary.

## Figures and Tables

**Table 1:** Physicochemical characterization of Mayten tree seed oil.

Analysis		Value
Color		
	L	53.94 ± 7.66 *
	a	24.92 ± 8.20 *
	b	41.02 ± 5.15 *
	C°	48.16 ± 8.34 *
	H°	59.40 ± 6.23 *
Density		1.06 ± 0.07 (g/mL)
Peroxide value		5.10 ± 0.18 (meq O_2_/kg oil)
Free Acidity		3.89 ± 0.19 (% oleic acid)
TBARS		5.74 ± 0.21(nmol/g lipids)
Rancimat		52.15 ± 2.15 (h)
Iodine value		57.63 ± 2.00 *

* adimentional value. All values reported in this table correspond to mean ± standard deviation.

**Table 2:** Composition of the relevant groups of fatty acids and the nutritional qualities of Mayten tree seed oil.

	Value
SFA	10.66 (g/100 g oil)
MUFA	25.70 (g/100 g oil)
PUFA	20.98 (g/100 g oil)
n-6	19.66 (g/100 g oil)
n-3	1.32 (g/100 g oil)
PUFA/SFA	1.97
n-6/n-3	12.62
AI	0.20
TI	0.07

**Table 3:** Phenolic and carotenoid compound contents in Mayten tree seed oil.

Parameter	Value
Total Phenolic content	16.1 ± 4.3 (mg GAE/100 g oil)
Total Flavonoids content	15.5 ± 2.3 (mg QE/100 g oil)
Total Carotenoids content	153.2 ± 6.5 (mg β-carotene E/100 g oil)
β-carotene content	106.8 ± 40.2 (mg β-carotene/100 g oil)
Coumaric acid	5.47 ± 0.03 (µg/100 g oil)
Quercetin	3.21 ± 0.05 (µg/100 g oil)
Myricetin	7.74 ± 0.04(µg/100 g oil)
Ferulic acid	49.64 ± 0.03 (µg/100 g oil)

All values reported in this table correspond to mean ± standard deviation.

**Table 4:** Antioxidants capacity in Mayten tree seed oil.

Properties	Value
Antioxidant Capacity-ABTS	1.25 ± 0.45 (mg TE/100 g of oil)
IC_50_	1.06 ± 0.03 (g oil)
FRAP	149 ± 7.45 (mg FeSO_4_/100 g of oil)

All values reported in this table correspond to mean ± standard deviation.

## Supplementary Materials

The following are available online at https://www.mdpi.com/article/10.3390/foods10040729/s1. Figure S1: (a) Fruits and seeds (orange-reddish color) of Mayten tree, (b) seed oil of Mayten tree extracted with solvents (methanol and chloroform).
