# Supplementary material for: Mayten Tree Seed Oil: Nutritional Value Evaluation According to Antioxidant Capacity and Bioactive Properties
Source: Foods. 2021 Mar 30;10(4):729. doi: 10.3390/foods10040729 (PMC8065577; doi:10.3390/foods10040729)
Supplement: Supplementary file 1 [file foods-10-00729-s001.pdf]

Supplementary material

Mayten tree and Mayten tree seed description

The Maitén (*Maytenus boaria*) is a tree species of the Celastraceae family, always green native to Chile, Argentina, Peru, and Brazil. It grows at the height of 5 m, and its wood can be used for foraging (Marcora, Tecco, Zeballos, & Hensen, 2017). In Chile, it adapts to different latitudes and altitudes being up to 1600 m above sea level, in different climatic conditions, availability of water resources and soil conditions, mainly in the north in ravines, river basins, and flood places; while in the central zone, they are distributed in the plains and the southern zone in the pre-cordilleran sector (Marcora et al., 2017).

Seeds inside have an endosperm, where their energy reserve is mostly based on fatty acids. Morphological studies have allowed the determination of the existence of specialized subcellular organelles in the endosperm cells of the Mayten seeds (oleosomes), being responsible for the storage of fatty acids (Godoy, 2017).

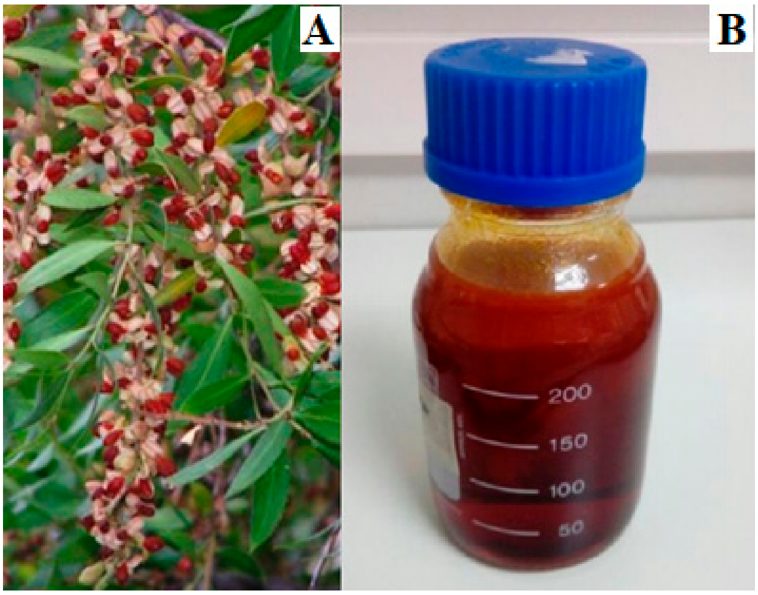


**Figure S1: A) Fruits and seeds (orange-reddish color) of Mayten tree, B) seed oil of Mayten tree extracted with solvents (methanol and chloroform).**
